# Potential impact of flooding on schistosomiasis in Poyang Lake regions based on multi-source remote sensing images

**DOI:** 10.1186/s13071-021-04576-x

**Published:** 2021-02-22

**Authors:** Jing-Bo Xue, Xin-Yi Wang, Li-Juan Zhang, Yu-Wan Hao, Zhe Chen, Dan-Dan Lin, Jing Xu, Shang Xia, Shi-Zhu Li

**Affiliations:** 1 National Institute of Parasitic Diseases, Chinese Center for Diseases Control and Prevention, Chinese Center for Tropical Diseases Research, Shanghai, 200025 People’s Republic of China; 2 WHO Collaborating Centre for Tropical Diseases, Shanghai, 200025 People’s Republic of China; 3 National Center for International Research on Tropical Diseases, Ministry of Science and Technology, Shanghai, 200025 People’s Republic of China; 4 Key Laboratory of Parasite and Vector Biology, Ministry of Health, Shanghai, 200025 People’s Republic of China; 5 School of Global Health, Chinese Center for Tropical Diseases Research, Shanghai Jiao Tong University School of Medicine, Shanghai, 200025 People’s Republic of China; 6 Jiangxi Institute of Parasitic Diseases, Nanchang, 330046 Jiangxi People’s Republic of China; 7 Jiangxi Key Laboratory of Schistosomiasis Prevention and Control, Nanchang, 330046 Jiangxi People’s Republic of China

**Keywords:** Schistosomiasis, Flood disaster, Remote sensing, Water extraction

## Abstract

**Background:**

Flooding is considered to be one of the most important factors contributing to the rebound of *Oncomelania hupensis*, a small tropical freshwater snail and the only intermediate host of* Schistosoma japonicum*, in endemic foci. The aim of this study was to assess the risk of intestinal schistosomiasis transmission impacted by flooding in the region around Poyang Lake using multi-source remote sensing images.

**Methods:**

Normalized Difference Vegetation Index (NDVI) data collected by the Landsat 8 satellite were used as an ecological and geographical suitability indicator of *O. hupensis* habitats in the Poyang Lake region. The expansion of the water body due to flooding was estimated using dual-polarized threshold calculations based on dual-polarized synthetic aperture radar (SAR). The image data were captured from the Sentinel-1B satellite in May 2020 before the flood and in July 2020 during the flood. A spatial database of the distribution of snail habitats was created using the 2016 snail survey in Jiangxi Province. The potential spread of *O. hupensis* snails after the flood was predicted by an overlay analysis of the NDVI maps in the flood-affected areas around Poyang Lake. The risk of schistosomiasis transmission was classified based on *O. hupensis* snail density data and the related NDVI.

**Results:**

The surface area of Poyang Lake was approximately 2207 km^2^ in May 2020 before the flood and 4403 km^2^ in July 2020 during the period of peak flooding; this was estimated to be a 99.5% expansion of the water body due to flooding. After the flood, potential snail habitats were predicted to be concentrated in areas neighboring existing habitats in the marshlands of Poyang Lake. The areas with high risk of schistosomiasis transmission were predicted to be mainly distributed in Yongxiu, Xinjian, Yugan and Poyang (District) along the shores of Poyang Lake. By comparing the predictive results and actual snail distribution, we estimated the predictive accuracy of the model to be 87%, which meant the 87% of actual snail distribution was correctly identified as snail habitats in the model predictions.

**Conclusions:**

Data on water body expansion due to flooding and environmental factors pertaining to snail breeding may be rapidly extracted from Landsat 8 and Sentinel-1B remote sensing images. Applying multi-source remote sensing data for the timely and effective assessment of potential schistosomiasis transmission risk caused by snail spread during flooding is feasible and will be of great significance for more precision control of schistosomiasis. 
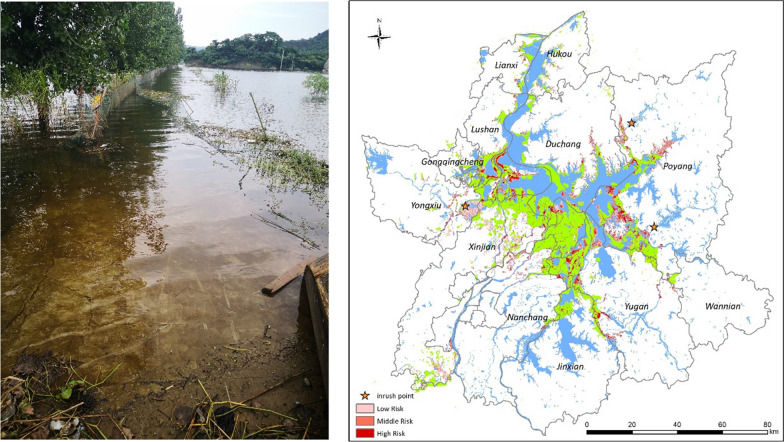

## Background

Intestinal schistosomiasis, caused by infection with *Schistosoma japonicum*, is a zoonotic parasitic disease [1]. In China, schistosomiasis is mainly concentrated along the south of the Yangtze River basin, where it has a substantial impact on human health and socioeconomic development [2, 3]. *Oncomelania hupensis*, a small tropical freshwater snail, is an amphibious species, and water is one of the essential conditions for its growth and reproduction; these snails will not thrive in persistently dry areas. Young snails live in the water, and mature snails generally live on wet and food-rich land. Regions where the water level varies significantly are suitable for snail breeding if the water flow is slow or the vegetation grows well. Variations in water level greatly affect the reproduction and growth of snails [4, 5].

*Oncomelania hupensis* is the only intermediate host of *S. japonicum.* [6]. Studies have shown that the geographical distribution of schistosomiasis is strongly associated with the distribution of *O. hupensis *[3]. The primary distribution of *O. hupensis* along the river system means that it plays a critical role in the transmission of schistosomiasis. In China, *O. hupensis* is predominantly distributed in marshland and lake areas along the middle and lower reaches of the Yangtze River in the provinces of Hunan, Hubei, Jiangxi, Anhui and Jiangsu. This area covers a landmass of 3.484 to 3.611 thousand km^2^ and accounts for a geographic distribution of 97.32–98.9% of snail habitats in China [7].

China began programs to eliminate schistosomiasis in 2016, and since then disease endemicity has been maintained at a historically low level in the country, with the exception of regions with extensive snail habitats [8]. Regular seasonal fluctuations in the water level of Poyang Lake create favorable conditions for snail breeding in the marshlands around this lake. Flooding in the area in 2017 contributed to the expansion of snail habitats along Poyang Lake [5, 9, 10] by 490 thousand km^2^, accounting for 98% of newly detected and re-emerging snail habitats in Jiangxi Province [11]. Additionally, infected bovines maintain a reservoir of schistosomiasis infection in the Poyang Lake region, acting as a source of onward transmission [12]. Recently, regions of schistosomiasis transmission have been identified by wild feces surveillance [13], detection of infected snails by loop-mediated isothermal amplification (LAMP) [14, 15] and sentinel mice-base surveillance [16].

Since June 2020, continuous heavy rainfall along the middle and lower reaches of the Yangtze River has resulted in severe flood disasters along the Yangtze River basin and in the areas around Poyang Lake. The highest recorded water level exceeded that of previous record highs following flooding in 1998, and has led to the collapse of dikes and/or waterlogging in many urban areas in China. Control of schistosomiasis in flood-affected areas has been impacted by increased contact with infested water during the fight against floods and the increased spreading of snails attributable to flooding. In this context, the aim of this study was to predict the expansion of *O. hupensis* snail habitats impacted by flooding and to assess the associated potential risk of schistosomiasis transmission in the areas around Poyang Lake using multi-source remote sensing image data and Sentinel-1B satellite radar images.

## Methods

### Study area

**Fig. 1:**
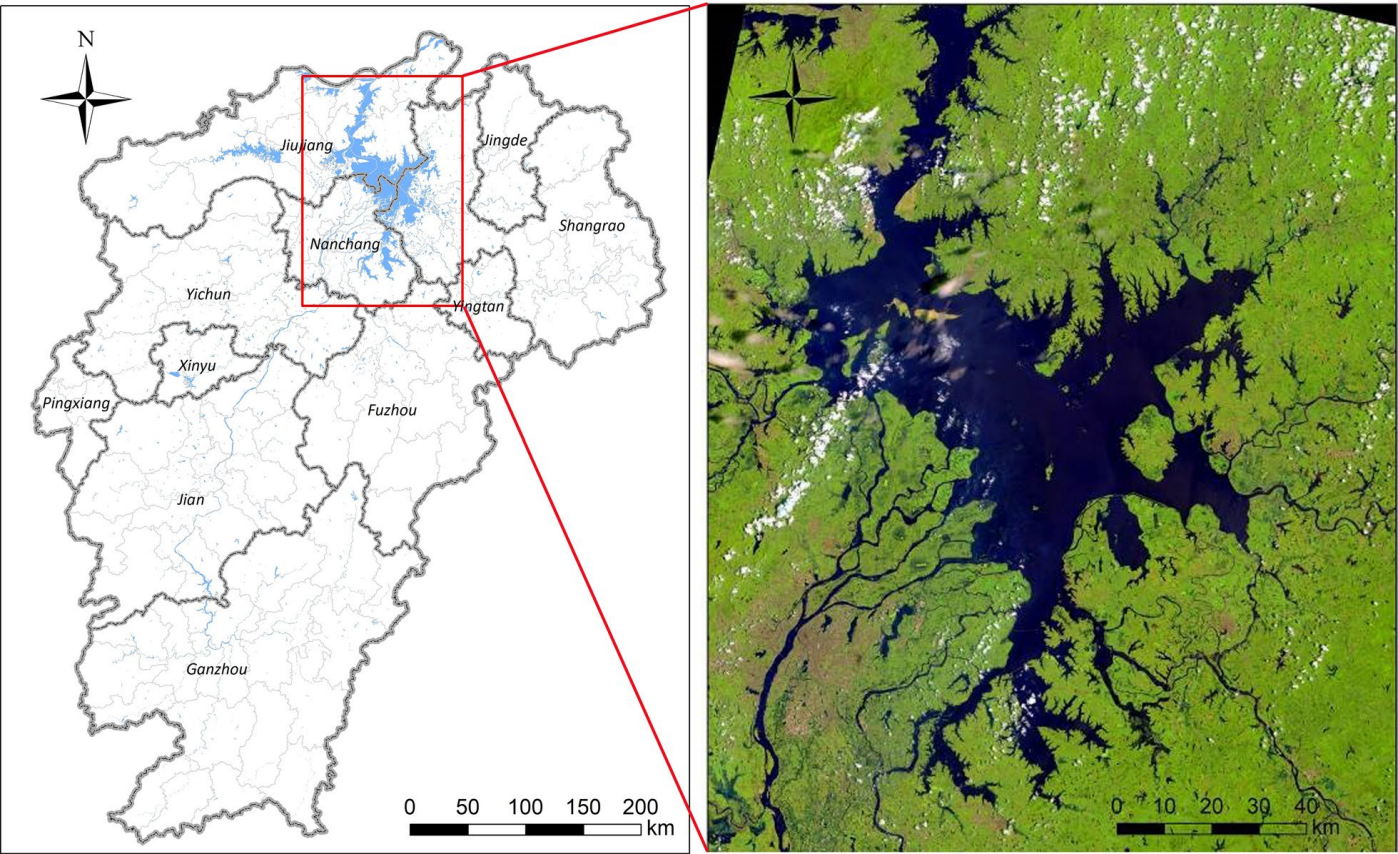
Geographical location of the study area. Poyang Lake is located in the north of Jiangxi Province and borders the middle and lower reaches of the Yangtze River. The natural geographical features of Poyang Lake are very suitable for snail breeding. A total of 13 counties (districts) in the areas around Poyang Lake, where schistosomiasis is prevalent, were included in this study

Poyang Lake, the largest freshwater lake in China, is located in the middle and lower reaches of the Yangtze River in the north of Jiangxi Province (28°22′–29°45′N,115°47′–116°45′E). The ecological and geographical features of Poyang Lake are suitable for snail breeding. The lake is fed by water from inland rivers between April and June and by the Yangtze River between July and September, which enables a high water level to be maintained during the spring and summer seasons. A total of 13 counties (cities, districts) in the areas around Poyang Lake are endemic for schistosomiasis, including Nanchang, Xinjian, Jinxian, the high-tech zone of Nanchang City, Yongxiu, Gongqingcheng, Lushan, Lianxi, Hukou, Duchang, Poyang, Yugan and Wannian (Fig. 1).

### Snail distribution

Data on snail distribution were obtained from a snail survey carried out in Jiangxi Province in 2016 [11]. The geographical and environmental characteristics of snail distribution were extracted for the 13 counties (cites, districts) in which schistosomiasis is endemic, and the spatial database of snail distribution in the Poyang Lake area was created accordingly. In this study, a 75% subset of the snail distribution data was randomly selected as a model training dataset, and the remaining 25% of the data was assigned as the model validation dataset.

### Multi-source remote sensing images

Remote sensing image data collected by the Landsat 8 satellite, with a spatial resolution of 30 m following geometric correction, convolution interpolation and resampling, were extracted from the NASA EarthData database (https://earthdata.nasa.gov/). Operational Land Imager (OLI) multi-wave remote sensing images were obtained for May 2016, the time period during which the snail survey was conducted. Sentinel-1B synthetic aperture radar (SAR) images were obtained from the European Space Agency (ESA) Earth Online database (https://earth.esa.int/), and remote sensing image data were obtained from the Sentinel-1B dual-polarized (HV + HH) SAR data for 15 May 2020, corresponding to the time period before the flood, and for 14 July 2020, corresponding to the period of peak flooding. An interferometric wide-swath mode was assigned as the image mode.

### Remote sensing image data inversion

Normalized Difference Vegetation Index (NDVI) data were used as a measure of vegetation coverage in snail habitats, calculated using the following formula [17]:

$$\mathrm{NDVI}=\frac{\mathrm{NIR}-R}{\mathrm{NIR}+R}$$

wherebNIR indicates the reflectance in near-infrared wavelengths, and *R* indicates the reflectance in visible red wavelengths. Maximum and minimum NDVI values for snail habitats in the areas around Poyang Lake were calculated based on a dataset of 75% of snail distribution sites [18, 19].

Areas where flood-caused expansion of the water body had occurred were identified using dual-polarized threshold calculations for the time period of the flooding [20]. SAR images were segmented by estimating the small backscattering coefficient thresholds of water on the SAR images [21]. Segmentation results were saved as classification result files, and the classification results were post-processed. Incorrect extraction images were removed through a human–computer interaction system to yield the final water body data. Sentinel-1B satellite data were processed using the SAR Scape module in the Environment for Visualizing Images (ENVI) software version 5.3 (Exelis Visual Information Solutions, Boulder, CO, USA), which mainly included radiometric calibration, filtering processing, terrain correction and geocoding. Radiometric calibration allows the transformation of image intensity into a backscattering coefficient (${\sigma }^{0}$), which was calculated using the following formula:

$${\sigma }^{0}=\frac{{A}^{2}}{K}sin\theta$$

where ${\sigma }^{0}$ is the backscattering coefficient of each pixel, *A* is the digital number of original images, *K* is an absolute calibration factor and $\theta$ is an incident angle.

Because the extraction of the water body data may be interfered with the intrinsic speckle noise in SAR images, a Frost filtering algorithm (5 × 5) was employed to control the output of a wave filter based on the local statistical characteristics of images [22]. The specific side-view mode of SAR images may lead to the occurrence of foreshortening, layover and shadow in mountains with terrain undulations, which affects the correct analysis of imaging data. Due to the occurrence of terrain undulations in the study area, terrain corrections of SAR images were performed to reduce the error in the water body data caused by geometric characteristics. Geocoding and radiometric calibration of filtered intensity data were also carried out using a digital elevation model (DEM) to generate backscattering coefficient images with dual-polarized (HV + HH) geographic coordinate systems. The index used for the water body data extraction was calculated based on the backscattering coefficients for HV and HH polarizations using the following formula:

$$\text{R}=\text{ln} \,(10\times \text{HV}\times \text{HH})$$

### Risk prediction of schistosomiasis transmission

Areas neighboring snail habitats which overlapped with flooded areas were extracted from SAR data to determine possible snail distribution zones following flooding [23]. Snail density was estimated from snail habitat-neighboring areas, and the NDVI values corresponding to snail distribution were calculated to predict potential snail spread and associated schistosomiasis transmission risk [24]. A 25% subset of observed snail breeding sites was selected as a validation dataset [19], and the distribution of snail breeding sites was predicted using model results in ArcGIS software version 10.1. Model predictions corresponding to the validation dataset were extracted, and the predictive accuracy of the model was assessed by identifying snail breeding sites correctly identified by the model [25].

## Results

### Distribution of* O. hupensis* snails in the areas around Poyang Lake

The results of a snail survey carried out in in Jiangxi Province in 2016 found snail habitats to be mainly distributed in 13 marshland and lake counties (cities, districts), including Nanchang, Xinjian, Jinxian, High-tech Zone of Nanchang City, Yongxiu, Gongqingcheng, Lushan, Lianxi, Hukou, Duchang, Poyang, Yugan and Wannian. A total of 1257 habitat settings were identified, with marshlands contributing to 74.94% of all identified habitat settings. Among the 763 snail habitats identified, 99.48% were marshland areas. In one marshland area covering 1267.57 km^2^, snail habitats accounted for 789.01 km^2^. Among these, along the northern shore of Poyang Lake 12 settings were identified with a density of living snails of ≥ 1 snail/0.1 m^2^ (7 settings in Lushan, 4 in Hukou and 1 in Yongxiu) and eight settings were identified with ≥ 50% occurrence of frames with living snails (5 settings in Lushan, 2 in Yongxiu and 1 in Hukou) (Table 1).

**Table 1:** Distribution of snail habitats in Poyang Lake areas

County	Total				Marshland				Ditch				Others			
	No. settings	No. settings with snails	Area of settings (km^2^)	Area of snail habitats (km^2^)	No. settings	No. settings with snails	Area of settings (km^2^)	Area of snail habitats (km^2^)	No. settings	No. settings with snails	Area of settings (km^2^)	Area of snail habitats (km^2^)	No. settings	No. settings with snails	Area of settings (km^2^)	Area of snail habitats (km^2^)
Nanchang	107	103	123.89	119.54	107	103	123.89	119.54	0	0	0	0	0	0	0	0
Xinjian	181	62	162.48	97.61	79	62	119.99	97.61	28	0	7.26	0	74	0	35.23	0
Jinxian	86	65	91.60	45.23	81	65	70.41	45.23	0	0	0	0	5	0	21.19	0
Nanchang High-tech Zone	16	1	22.20	0.12	16	1	22.20	0.12	0	0	0	0	0	0	0	0
Lianxi	58	35	37.86	15.31	41	32	35.03	14.89	12	3	2.24	0.42	5	0	0.60	0
Hukou	102	18	49.20	2.27	29	18	14.24	2.27	6	0	1.56	0	67	0	33.40	0
Yongxiu	39	39	50.12	50.12	39	39	50.12	50.12	0	0	0	0	0	0	0	0
Duchang	253	183	421.87	145.06	217	183	421.87	145.06	0	0	0	0	36	0	0	0
山Lushan	79	53	105.53	54.21	60	52	92.75	54.09	4	1	1.06	0.12	15	0	11.71	0
Gongqingcheng	58	22	16.13	6.06	57	22	16.13	6.06	1	0	0	0	0	0	0	0
Poyang	145	111	184.98	137.49	144	111	184.18	137.49	1	0	0.80	0	0	0	0	0
Yugan	122	71	116.54	116.54	71	71	116.54	116.54	0	0	0	0	51	0	0	0
Wannian	11	0	2.26	0	1	0	0.23	0	0	0	0	0	10	0	2.03	0
Total	1257	763	1384.65	789.55	942	759	1267.57	789.01	52	4	12.92	0.54	263	0	104.16	0

### Environmental identification for suitable snail habitats

**Fig. 2:**
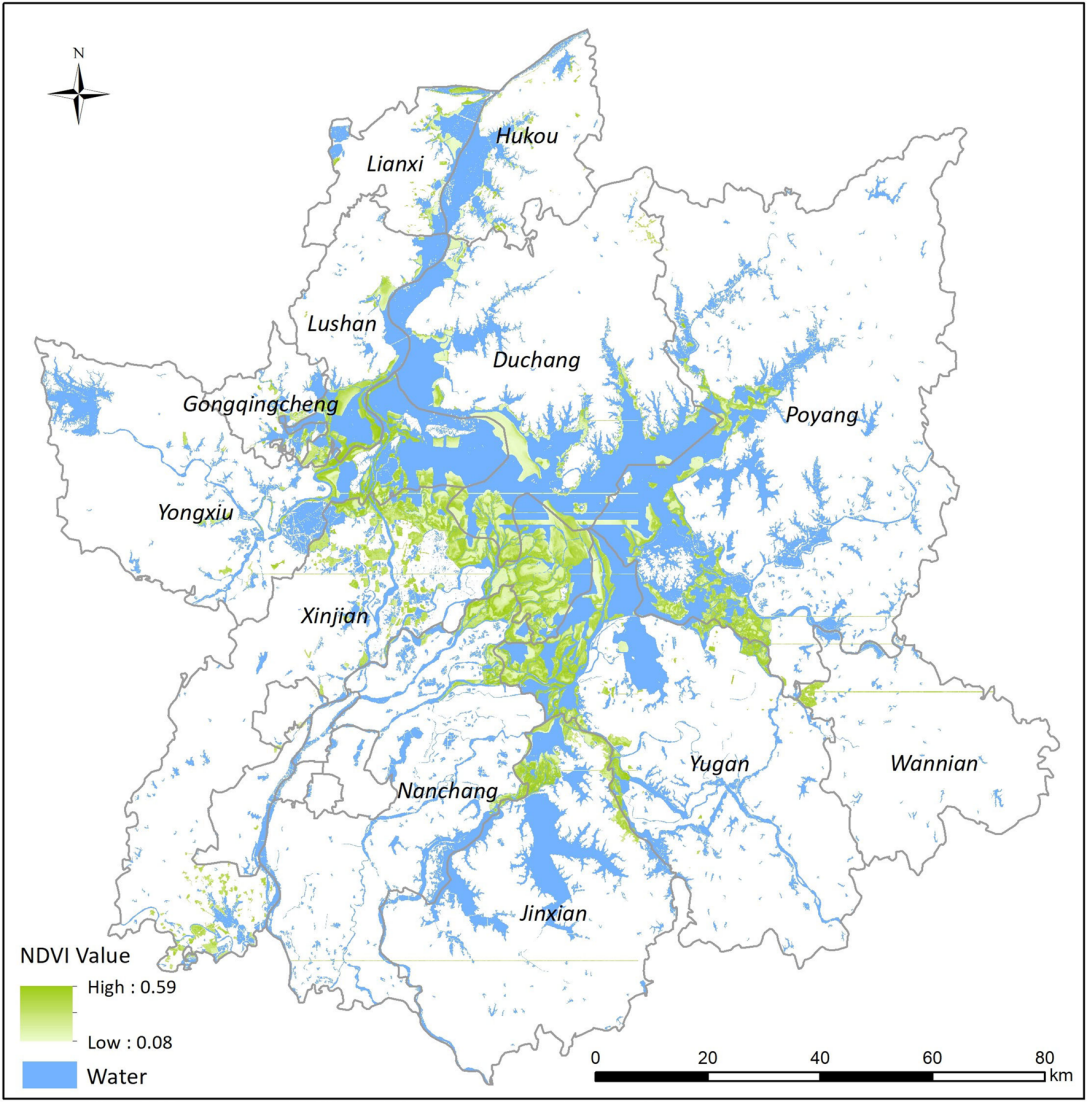
Distribution of snail habitats in Poyang Lake areas. Snail data were captured from the 2016 snail survey in Jiangxi Province. Normalized Difference Vegetation Index (*NDVI*) data were used to measure the vegetation coverage in snail habitats, collected from Landsat 8 satellite remote sensing images from the NASA EarthData database

The NDVI values were estimated to range from − 1 to 0.61 in the Poyang Lake area based on Landsat 8 remote sensing image data. A total of 75% of snail distribution sites were randomly sampled from snail habitats identified by field surveys as a training dataset to extract the optimum thresholds of the maximum and minimum value of NDVI at 95% confidential interval (C)] 0.08–0.59. The distribution of suitable snail habitats is shown in Fig. 2.

### Extraction of flood-cased water body expansion

**Fig. 3:**
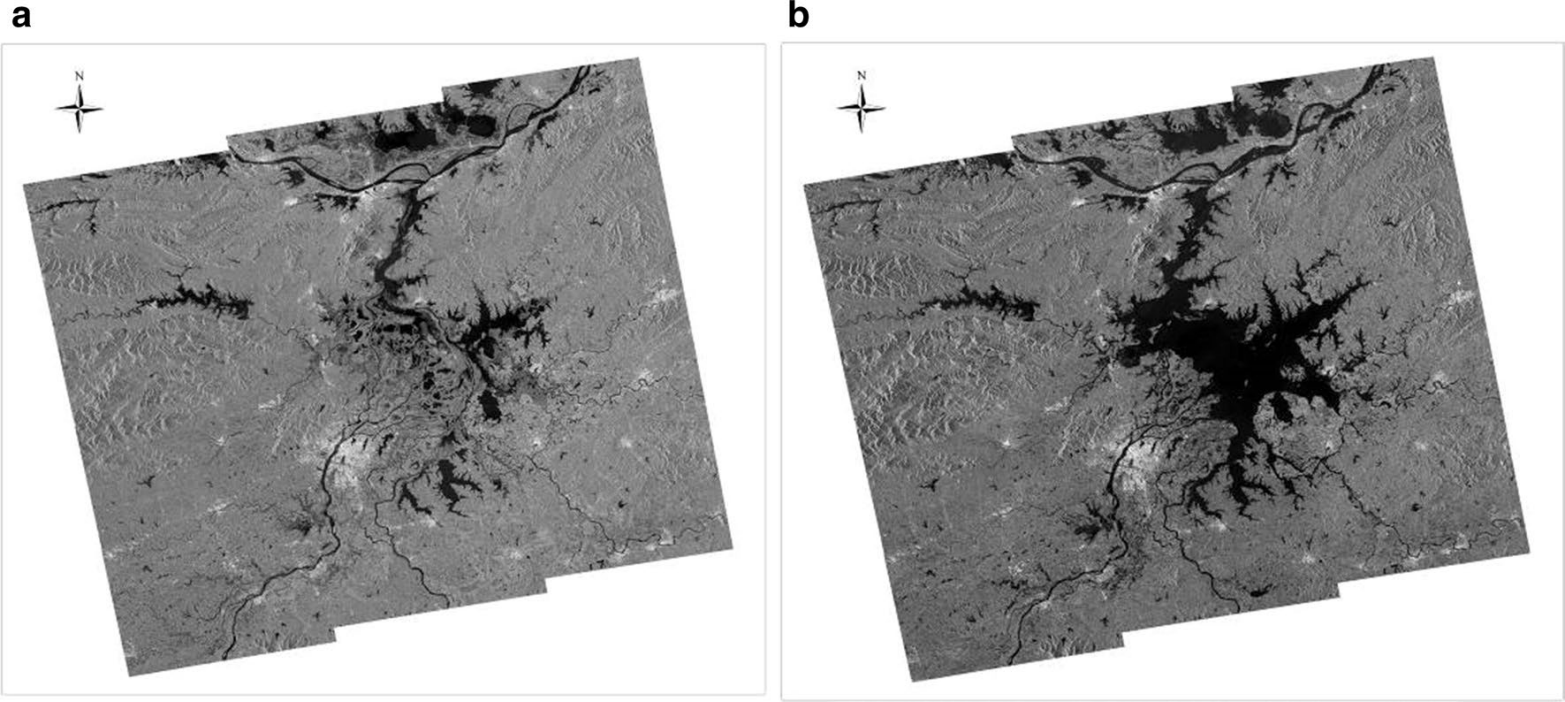
Waterbody extraction from remote sensing image data in Poyang Lake areas. Image data were collected from ESA Earth Online database of Sentinel-1B dual-polarized (HV + HH) SAR data on 15 May 2020, before the flood (**a**), and on 14 July 2020, during the period of the flood peak (**b**)

Radar echo intensity was determined by brightness in the SAR data. Due to the low echo intensity of water bodies and high echo intensity of the corresponding land areas, water body areas in SAR images appeared as dark or black and the land areas as grayish white or dark gray. Pre-processed SAR images from Sentinel-1B satellite images for the time period before and after flooding are shown in Fig. 3. The speckle noise was effectively inhibited, and a more obvious water–land boundary was seen on original radar images in which the water and land were well differentiated and the water profile was more distinct.

**Fig. 4:**
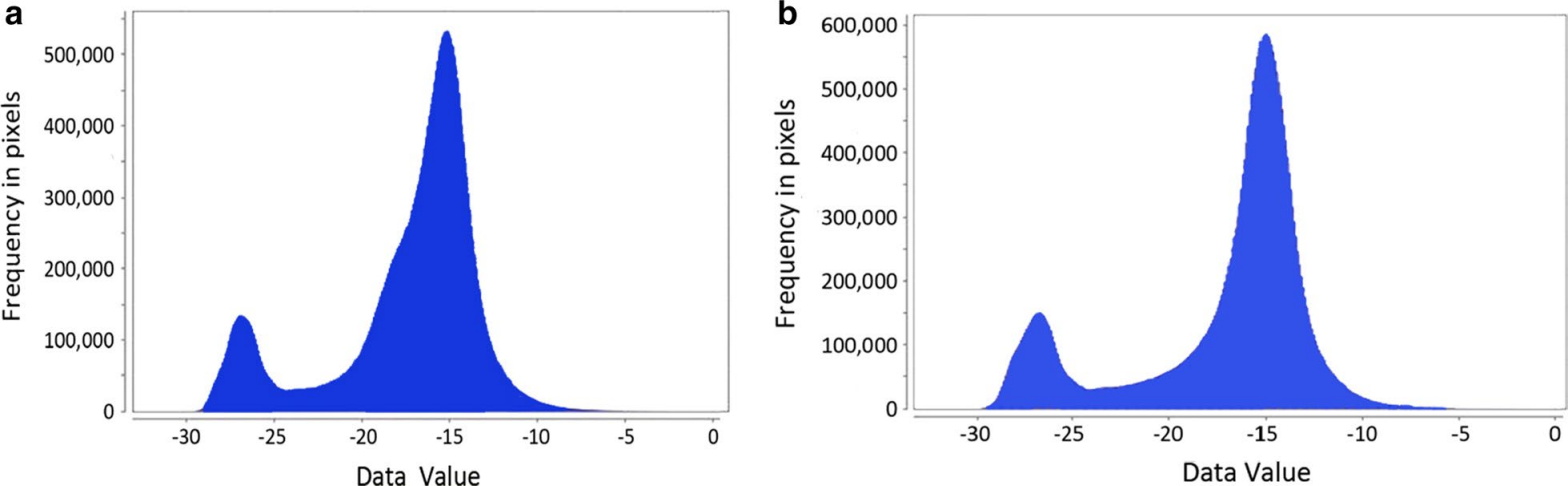
The histogram of scattering values of SAR radar images for waterbody extractions. **a** An image captured on 15 May 2020, **b** an image captured on 14 July 2020. There are two apparent peaks in the histograms of the images, and the thresholds for segmented SAR images were − 25.5 and − 24 dB before and after flooding, respectively

Figure 4 shows a histogram of pre-processed SAR image scattering values before and after flooding. There are two apparent peaks in the histograms in the images presented in Fig. 4, with segmented SAR image thresholds ranging from − 25.5 dB (before flooding) to − 24 dB(after flooding) based on visual interpretation.

Segmentation results were saved as classification results and transformed to a vector file. Following post-classification data processing, incorrect extraction images were removed through a human–computer interaction to generate a final water body dataset for the Poyang Lake area for the time periods of May 15 and July 14.

### Changes in water body areas before and after flooding

**Fig. 5:**
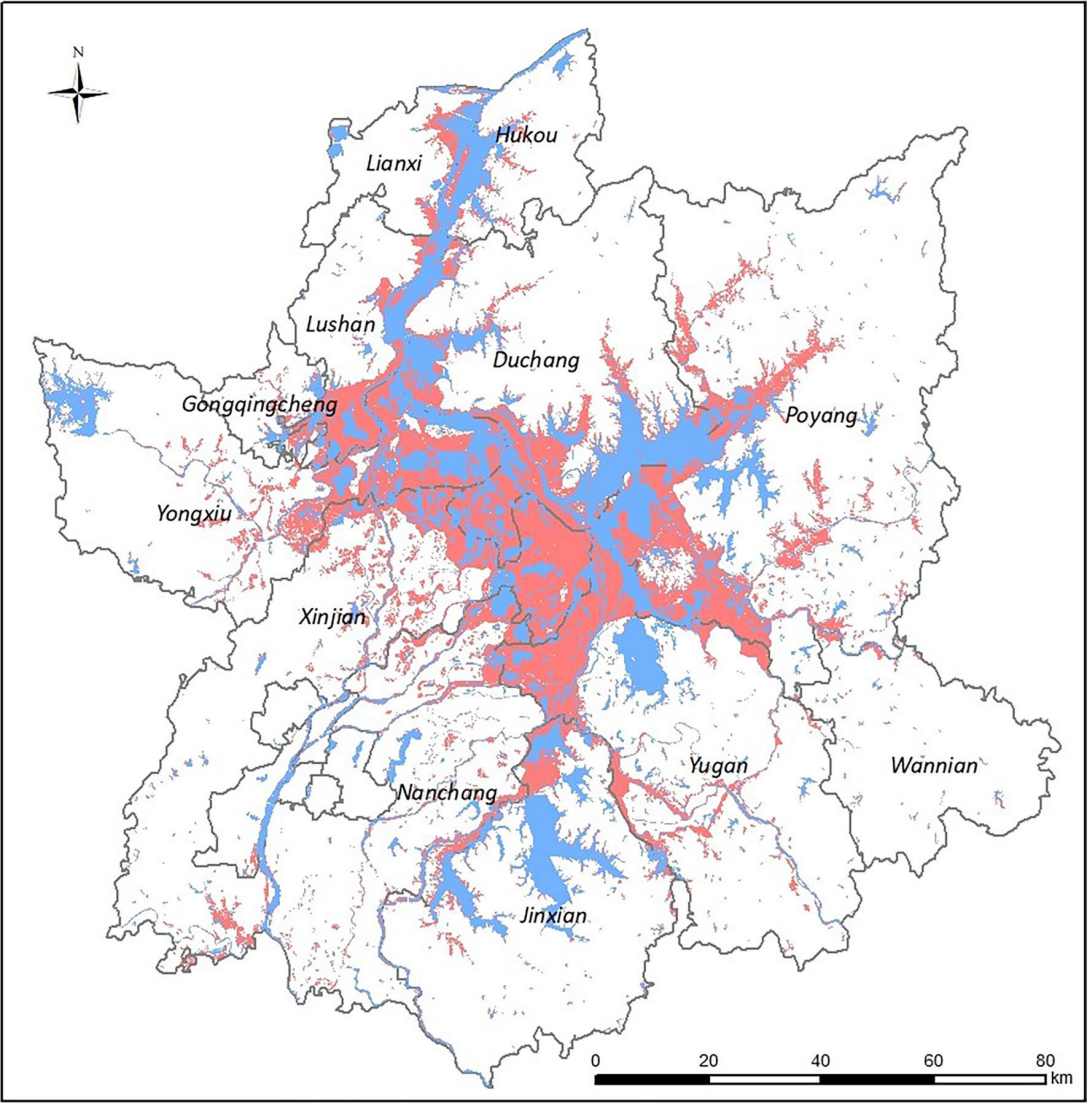
Changes in the area of water bodies before and after flooding in the Poyang Lake areas. The blue regions indicate the expanse of water bodies on 15 May 2020, before the flood, and the red regions indicate the expansion in the area of the water bodies on 15 July, during the flood period

The distribution of water bodies in areas around Poyang Lake was compared by overlapping imaging data before and after flooding. The blue areas in Fig. 5 indicate the distribution of water bodies before the flood, and red areas describe the expansion of water bodies during flooding. Examination of the main body of Poyang Lake and neighboring water areas from Sentinel-1B SAR images showed that approximately 2207 km^2^ was covered by water on May 15 and that 4403 km^2^ was covered by water on July 14, an increase of 2196 km^2^ compared to May and an increase of 25.4% compared to the historical mean level during the same period (3510 km^2^). Taken as a whole, the area covered by water expanded by approximately 99.5% after flooding relative to the areas covered by water before flooding, with the increase occurring primarily in Xinjian, Duchang, Poyang, Yongxiu and Yugan (Fig. 5).

### Risk Predictions of potential snail diffusion and associated schistosomiasis transmission after the flood

**Fig. 6:**
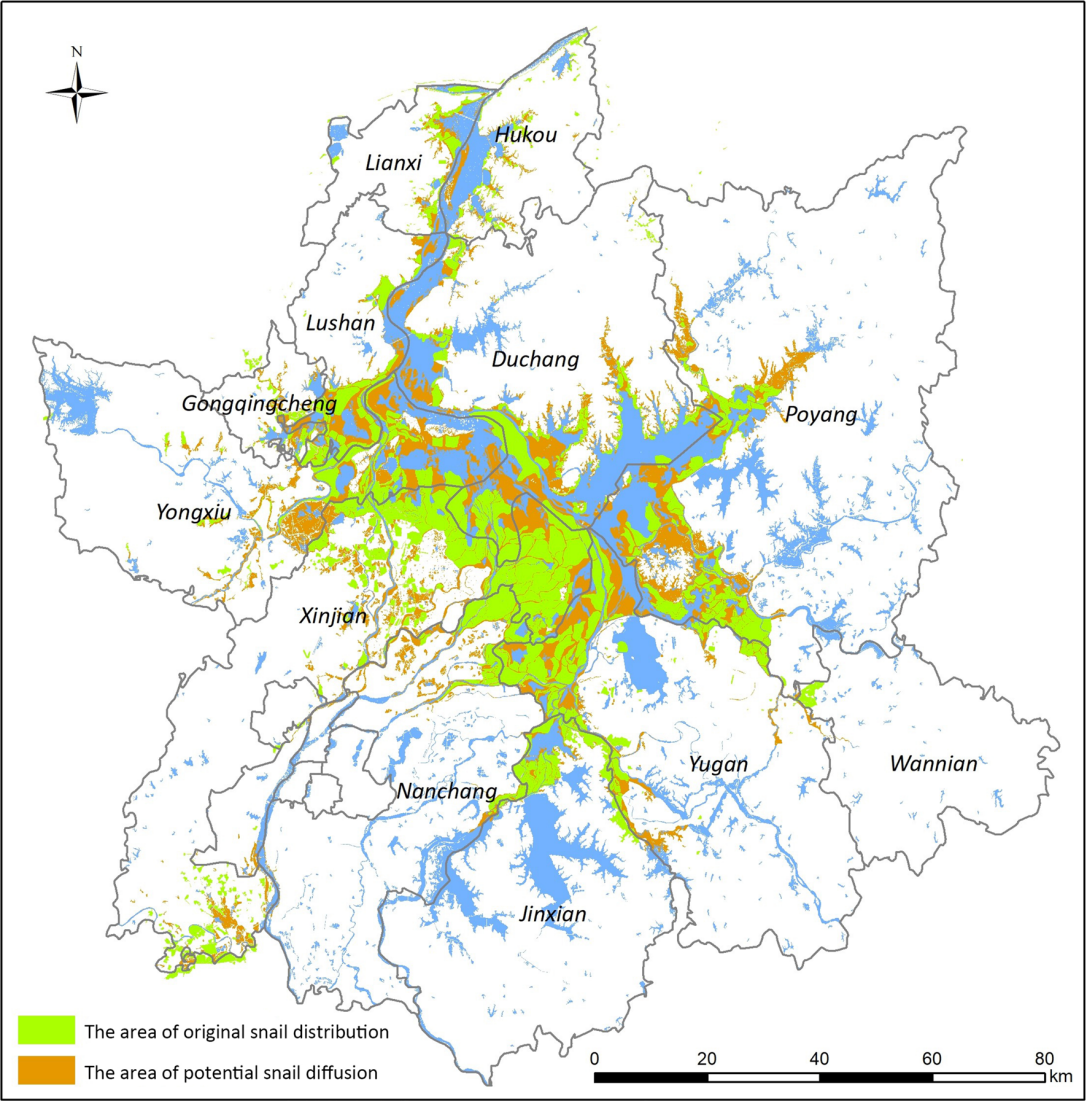
Prediction of snail habitats based on flood-affected settings. Snail spread was predicted to possibly cover an area of approximately 759 km^2^ and to mainly ocuur east of Yongxiu, south of Lushan, southwestern Poyang, southwestern Duchang, northwestern Xinjian and northwestern Yugan

Data indicating flood-affected water areas were transformed into a binary image of potential snail habitat distribution. Areas of predicted snail diffusion exhibited a patchy, clustered distribution. After submersion of snail habitats following flooding, snail habitats were likely to be in neighboring settings. Snail distribution was predicted to cover an area of approximately 759 km^2^, mainly occurring in the east of Yongxiu, south of Lushan, southwestern Poyang, southwestern Duchang, northwestern Xinjian and northwestern Yugan. This predicted distribution suggested that areas of possible snail diffusion were predominately concentrated in marshlands around Poyang Lake (Fig. 6).

**Fig. 7:**
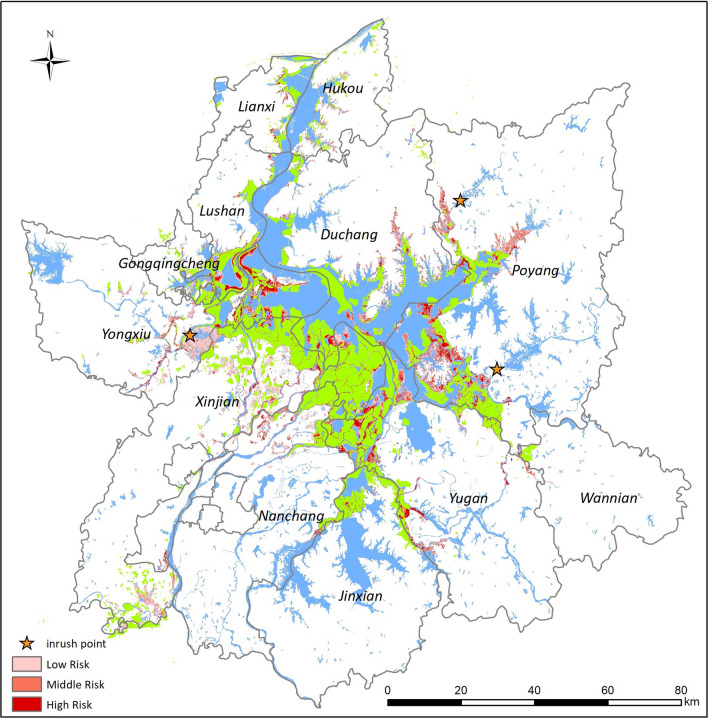
Risk classification of spread of *Oncomelania hupensis*. The NDVI values of suitable snail habitats were calculated based on snail density data, and the risk was classified as high-density, medium-density and low-density snail habitats with reference to the NDIV values

NDVI values of suitable snail habitats were calculated based on snail density data, with values ranging from 0.15 to 0.35 in high-density snail habitats, from 0.35 to 0.42 in medium-density snail habitats and from 0.08–0.15 to > 0.42 in low-density snail habitats. NDVI values of flood-affected areas were estimated and the risk of potential snail spread classified accordingly. We found that areas at high risk of snail distribution were predominantly located in northwestern Yongxiu, southwestern Duchang, south of Lushan and southwestern Poyang (Fig. 7). These high-risk areas are also indicative of neighboring areas suitable for snail breeding where snail habitats are likely to emerge following flooding, with the potential for schistosomiasis transmission. Validation of predicted snail habitats was carried out using observational data on snail breeding habitats in order to assess the predictive performance of the model’s NDVI value of snail habitats, which ranged from 0.1 to 0.52 using the 25% validation dataset, with 87% prediction accuracy, indicating that the NDVI values are a good predictor of snail habitats.

Multiple dikes collapsed following the flooding event in Jiangxi Province. Three sites in Yongxiu and Poyang counties where dikes had collapsed were found to overlap with areas classified as snail distribution risk areas. The sites in Yongxiu and Poyang County where dykes collapsed were predicted to be medium-risk areas of snail distribution, (Fig. 7). The data suggested that snail habitats were likely to emerge at both sites.

## Discussion

Schistosomiasis is a neglected tropical parasitic disease that is strongly associated with ecological and geographic factors. Changes in the natural environments which affect the breeding, reproduction and distribution of intermediate host snails are likely to impact the transmission of schistosomiasis [26, 27]. Previous research has found snail distribution to be closely correlated with vegetation, humidity and temperature, as well as with human and livestock activities, with *O. hupensis* snails favoring marshlands, ponds and ditches. As the geographical location of schistosomiasis is determined by snail distribution, surveillance of snail habitats is an important component in the national schistosomiasis control program in China.

Flooding frequently occurs along the south and middle and lower reaches of the Yangtze River, areas which are endemic for schistosomiasis. Flooding is considered to be one of the most important natural factors impacting the rebound of schistosomiasis in endemic foci. Periods of flooding impact the geographical expanse, reproduction and growth of *O. hupensis* snails, particularly juvenile snails and snail eggs, which spread via water flow [4, 28, 29]. Additionally, flood discharge or dike collapse may facilitate the expansion of snail populations into embankments, resulting in the re-emergence of snails in areas where snails had not previously been present [24].

The endemicity of schistosomiasis in China is currently declining, with the country progressing towards elimination [12]. Many challenges to schistosomiasis elimination remain, however, due to natural, biological and social factors [8]. Prevalence of schistosomiasis is high in 11 of the 13 counties (districts) in the Poyang Lake area in Jiangxi Province, with the exception of the high-tech zone of Nanchang City and Wannian County. In 2018, three egg-positive individuals were identified in Yugan County (in total, 8 egg-positive cases in China) and two egg-positive bovines were identified in Duchang County (in total, 2 egg-positive bovines in China) in 2019, suggesting that the risk of schistosomiasis transmission remains in the Poyang Lake area. Interestingly, these areas were identified as high-risk transmission areas in the present study. In areas characterized by vast marshlands, multiple grasslands, dense vegetation, difficulty in managing water levels and extensive snail distribution where schistosomiasis was once hyper-endemic and transmission had been controlled or interrupted [9], *S. japonicum* transmission potential remains due to the distribution of *O. hupensis* snails following flooding. Any relaxing of control interventions would therefore likely result in re-emergence of schistosomiasis infection.

Remote sensing image data have been widely employed for the surveillance of schistosomiasis and the habitats of the intermediate host snails [24, 30–33]. Satellite-based remote sensing collects ecological and geographical data, such as land coverage, vegetation, soil type, surface moisture and rainfall, which may be used to monitor environmental changes, thereby enabling assessment of the suitability of snail breeding habitats to have association potential for schistosomiasis transmission [25, 34–36]. Some limitations are inherent in data of this type, however, such as a short wavelength and potential effects of cloud amount and severe weather. Most notably, during flooding continuous cloudy and rainy conditions may lead to failure in the accurate acquisition of image data in flood-affected areas. Radar images, characterized by full-time and full-weather, high-coverage, high-resolution observations and high revisit rate, are not affected by meteorological conditions or light levels and have been widely employed in the fields of disaster monitoring, agriculture and oceanography [37–42].

In the current study, the distribution of snail habitats was estimated using Landsat 8 satellite remote sensing image data, and areas where water body overlapped before and after flooding were identified using Sentinel-1B dual-polarized image data. The total water body area was estimated to have expanded by approximately 99.5% in the Poyang Lake region after flooding relative to the water body area before flooding. Snail habitats are likely to emerge in flood-affected areas after flooding has receded. All of these regions were found to be adjacent to, or connected with, original snail habitats, predominantly distributed to the southwest and northwest of Poyang Lake. Snail distribution was predicted to mainly occur in the counties of Yongxiu, Lushan, Poyang, Duchang and Yygan and in Xinjian District. Based on these data, it can be predicted that multiple regions at high risk of schistosomiasis transmission are located in the counties of Yongxiu, Duchang, Lushan and Poyang. The classification of potential snail habitats is consistent with the spatial distribution of schistosomiasis transmission risk in the Poyang Lake regions based on schistosomiasis case reports [9].

## Conclusion

Remote sensing techniques were found to be an effective quantitative tool for the rapid assessment of snail distribution, providing insights into schistosomiasis risk and thereby providing valuable information for control programs. It is recommended that monitoring of *O. hupensis* and epidemiological surveys of *S. japonicum* infections should be conducted by schistosomiasis control institutions in the coming 2–3 years, with the aim to prevent the spreading of snail populations and to reduce the risk of transmission of schistosomiasis.

## Data Availability

The datasets analyzed during the present study are available from the corresponding authors upon reasonable request.

## Abbreviations

DEM: Digital elevation model
ENVI: Environment for visualizing images
LAMP: Loop-mediated Isothermal Amplification
NDVI: Normalized Difference Vegetation Index
OLI: Operational land imager
SAR: Synthetic aperture radar
